# Biological Membranes as Targets for Natural and Synthetic Compounds

**DOI:** 10.3390/membranes12121172

**Published:** 2022-11-22

**Authors:** Luis Octavio Regasini

**Affiliations:** Head of Laboratory of Antibiotics and Chemotherapeutics, Department of Chemistry and Environmental Sciences, Institute of Biosciences, Humanities and Exact Sciences, São Paulo State University, Rua Cristóvão Colombo 2265, São José do Rio Preto 15054-000, Brazil; luis.regasini@unesp.br

Biological membranes are responsible for all types of regulation and compound transfer, as well as information flow between and within eukaryotic and prokaryotic cells. For example, the plasma membrane is involved in both the generation and receipt of chemical and electrical signals; cell adhesion, which is responsible for tissue or biofilm information; cell locomotion; biochemical reactions; and cell reproduction. Internal membranes regulate a myriad of activities in organelles such as endosomes and the mitochondria. In this context, membranes play a key role in maintaining cell integrity, and their involvement in cellular function makes these regions of cells potential targets for bioactive and therapeutic compounds.

This *Membranes* Special Issue is devoted to state-of-the-art research on topics concerning the discovery and development of natural and synthetic compounds that act on biological membranes. The Special Issue contains seven elegant articles—six research articles and one review—with complete descriptions of each investigation and the main results presented in full respective manuscripts, which the readers are invited to read. A summary of the articles is presented herein.

The first research article of this Special Issue, by Kleinwächter et al. [1], showed that 2-phenylethanol (2-PEtOH) and its derivatives—phenylacetic acid, phenyllactic acid, and methyl phenylacetate—were fully incorporated into model membranes, affecting membrane organization. To estimate the membrane-binding affinity, the authors calculated the log *P* values of 2-PEtOH and its derivatives using Molinspiration Cheminformatics software (version 2016.10). The bacteriostatics of alcohols were evaluated against *Escherichia coli*, establishing the minimal inhibitory concentration able to inhibit 50% bacterial growth. A linear correlation between log *P* and log (1/MIC_50_) was described with a correlation coefficient (r^2^) value of 0.987. In order to clarify the effects of 2-PEtOH and its derivatives, in silico simulations indicated the intercalation orientations of compounds into membranes.

The article by Andrade-Ochoa et al. [2] presented the biological evaluation and chemical composition of essential oils (EOs) from anise, cinnamon, clove, cumin, laurel, lime, and oregano. The EOs were tested against a large panel of Gram-positive and Gram-negative bacteria, filamentous fungi, and protozoa. Antimicrobial assays, evidenced as minimal inhibitory concentration (MIC) values, suggested that oregano essential oil was the most potent antibacterial agent (MIC = 66–100 µg mL^−1^), while cinnamon essential oil had the highest antifungal activity (MIC = 66–116 µg mL^−1^). The most potent antiprotozoal actions were assigned to oregano and cinnamon EOs, with the IC_50_ and LD_50_ values ranging from 22 µg mL^−1^ to 108 µg mL^−1^, including the effects against *Trypanosoma cruzi*, *Leishmania mexicana*, and *Giardia lamblia*. Major compounds of EOs were also evaluated, and the most active ones were thymol, carvacrol, and cinnamaldehyde. A highlight of this article was the use of principal component analysis (PCA) to demonstrate the relationship between bioactivity of EOs and analyzed microorganisms, as well as the bioactivity of major compounds on these microorganisms.

In the study of Liu et al. [3], the authors investigated the role of an evolutionarily conserved multidrug resistance protein (MRP) in metabolic homeostasis by knocking down the expression of *Drosophila* multidrug resistance such as protein 1 in several sites related to the regulation of insect metabolism, such as gut, fat body, and Malpighian tubules. Despite many studies being dedicated to different organs, only the suppression of *MRP* in Malpighian tubules was reported to have significant effects; this organ is functionally similar to the human kidney. The reduction in Malpighian tubule *MRP* expression caused abnormal lipid accumulation and the disruption of feeding behavior. In addition, the *MRP* suppression led to an increase in the expression of *Hr96* (homolog of human *pregnane X receptor*), which acts in detoxication and lipid metabolism processes. Reduced expression of *MRP* in the Malpighian tubules also conveyed resistance to oxidative stress, as well as reduced normal levels of reactive oxygen species in adult flies. Altogether, this article revealed that an evolutionarily conserved MRP is required in *Drosophila* Malpighian tubules for proper metabolic homeostasis, providing a new insight to guide investigations of MRP in metabolic homeostasis.

In their original research article, Elexpe et al. [4] explore the oxidative damage caused by antimalarial drugs and plant EOs. They developed cell membrane microarrays, and superoxide production was evoked by intense mitochondrial activity in the presence of specific inhibitors of the mitochondrial electron transport chain, including rotenone, antimycin A, and azide. A protocol was established using membranes from rat brain regions and the effects of atovaquone, quinidine, doxycycline, mefloquine, artemisinin, tafenoquine, and EOs of *Rosmarinus officinalis* and *Origanum majoricum* were investigated in multiple human samples. The basal activity was related to the type of tissue, and the liver, jejunum, and adrenal gland exhibited the highest superoxide accumulation. The antimalarials showed specific behaviors according to the nature of the human tissue. Atovaquone and quinidine displayed the highest percentage of superoxide production and doxycycline the lowest. In summary, the superoxide production in cell membranes of a panel of human tissues allowed for the characterization of the safety profile of selected antimalarial drugs and EOs against toxicity mediated oxidative stress.

Doltchinkova, Mouleshkova, and Vitkova [5] studied the effects of excitatory neurotransmitter L-glutamic acid and its agonist kainic acid on Na^+^,K^+^-ATPase and Mg^2+^-ATPase activities in synaptic membranes prepared from the cerebral cortex of rat brain tissue. The surface parameters of synaptosomes in the presence of L-glutamic and kainic acids were characterized by microelectrophoresis. The acids promoted a significant increase in the electrophoretic mobility and surface electrical charge of synaptosomes at 1–4 h after isolation. The decrease in the bending modulus of model bimolecular membranes composed of monounsaturated lipid 1-palmitoyl-2-oleoyl-sn-glycero-3-phosphocholine provided evidence for softer membranes in the presence of L-glutamic acid. Kainic acid did not affect membrane mechanical stability, even at 10-fold higher concentrations. L-glutamic and kainic acids were able to reduce acetylcholinesterase activity and deviation from the normal functions of neurotransmission in synapses. Altogether, the acquired results manifested the modulation of the electrokinetic properties of synaptosomes and enzyme activity of synaptic membranes from the rat brain cortex cerebral upon the action of glutamate and kainate. In conclusion, this article, regarding the modulation of the enzyme activity of synaptic membranes and surface properties of synaptosomes, should inspire biochemical and biophysical investigations, contributing to the elucidation of the molecular mechanisms of neurotransmitters and their agonists on membranes.

The final research article in this Special Issue, by Assis et al. [6], described the synthesis and antibacterial activity of isobavachalcone (IBC) against a panel of Gram-positive, Gram-negative, and *Mycobacterium* species. IBC was active against Methicillin-Susceptible *Staphylococcus aureus* (MSSA) and Methicillin-Resistant *Staphylococcus aureus* (MRSA), with MIC values of 1.56 and 3.12 µg mL^−1^, respectively. However, IBC was not able to act against Gram-negative species (MIC > 400 µg mL^−1^). IBC displayed an effect against *Mycobacterium tuberculosis*, *Mycobacterium avium*, and *Mycobacterium kansasii* (MIC = 64 µg mL^−1^). IBC was able to inhibit more than 50% of MSSA and MRSA biofilm formation at 0.78 µg mL^−1^. Its antibiofilm activity was similar to vancomycin, which was active at 0.74 µg mL^−1^. In order to study the mechanism of IBC action by fluorescence microscopy, propidium iodide (PI) and SYTO9 fluorophores indicated that IBC disrupted the membrane of *Bacillus subtilis*. Toxicity assays using human keratinocytes (HaCaT cell line) showed that IBC did not have capacity to reduce the cell viability. In conclusion, the authors suggested that IBC is a promising antibacterial agent with an elucidated mode of action and potential applications as an antibacterial drug and a medical device coating.

The review by Jasni et al. [7] is an overview of compounds able to act against *Entamoeba histolytica*, the causative agent of amoebiasis. The article focuses on natural and synthetic antiprotozoals, as well as their molecular targets, including protozoan membranes, and proteins, including thioredoxin reductase, cysteine protease, phosphatases, triosephosphate isomerase, alcohol dehydrogenase, GTPases, KERP1, kinases, Niemann Pick Type (NPC), interferon-gamma receptor, and ERGIC53-like protein.

In general, the studies of synthetic and natural compounds involve proteins as targets. On the other hand, few bioactive compounds target membranes. In this Special Issue, we have collected relevant contributions on different classes of compounds and their correlations with biomembranes. Moreover, these contributions also highlight the high impact of the use of membranes to design innovative protocols useful to drug discovery. In conclusion, the objectives of this Special Issue have been successfully achieved; for this reason, I would like to express my heartfelt appreciation to all the authors, reviewers, and editors involved for their excellent contributions.

## Data Availability

Not applicable.
